# Comparison of clinical efficacy between LAPS and ALPPS in the treatment of hepatitis B virus-related hepatocellular carcinoma

**DOI:** 10.1093/gastro/goad060

**Published:** 2023-10-12

**Authors:** Zebin Chen, Shunli Shen, Wenxuan Xie, Junbin Liao, Shiting Feng, Shaoqiang Li, Jiehui Tan, Ming Kuang

**Affiliations:** Centre of Hepato-Pancreato-Biliary Surgery, The First Affiliated Hospital, Sun Yat-sen University, Guangzhou, Guangdong, P. R. China; Centre of Hepato-Pancreato-Biliary Surgery, The First Affiliated Hospital, Sun Yat-sen University, Guangzhou, Guangdong, P. R. China; Centre of Hepato-Pancreato-Biliary Surgery, The First Affiliated Hospital, Sun Yat-sen University, Guangzhou, Guangdong, P. R. China; Centre of Hepato-Pancreato-Biliary Surgery, The First Affiliated Hospital, Sun Yat-sen University, Guangzhou, Guangdong, P. R. China; Department of Diagnostic Radiology, The First Affiliated Hospital, Sun Yat-sen University, Guangzhou, Guangdong, P. R. China; Centre of Hepato-Pancreato-Biliary Surgery, The First Affiliated Hospital, Sun Yat-sen University, Guangzhou, Guangdong, P. R. China; Department of Hepatobiliary Surgery, The Third Affiliated Hospital, Sun Yat-sen University, Guangzhou, Guangdong, P. R. China; Centre of Hepato-Pancreato-Biliary Surgery, The First Affiliated Hospital, Sun Yat-sen University, Guangzhou, Guangdong, P. R. China

**Keywords:** LAPS, ALPPS, hepatitis B virus, hepatocellular carcinoma

## Abstract

**Background:**

Insufficient post-operative future liver remnant (FLR) limits the feasibility of hepatectomy for patients. Staged hepatectomy is an effective surgical approach that can improve the resection rate of hepatocellular carcinoma (HCC). This study aimed to compare the safety and efficacy of laparoscopic microwave ablation and portal vein ligation for staged hepatectomy (LAPS) and classical associating liver partition and portal vein ligation for staged hepatectomy (ALPPS) in the treatment of hepatitis B virus (HBV)-related HCC.

**Methods:**

Clinical data of patients with HBV-related HCC who underwent LAPS or ALPPS in our institute between January 2017 and May 2022 were retrospectively analysed.

**Results:**

A total of 18 patients with HBV-related HCC were retrospectively analysed and divided into the LAPS group (*n *=* *9) and ALPPS group (*n *=* *9). Eight patients in the LAPS group and eight patients in the ALPPS group proceeded to a similar resection rate (88.9% vs 88.9%, *P *=* *1.000). The patients undergoing LAPS had a lower total comprehensive complication index than those undergoing ALPPS but there was not a significant different between the two groups (8.66 vs 35.87, *P *=* *0.054). The hypertrophy rate of FLR induced by ALPPS tended to be more rapid than that induced by LAPS (24.29 vs 13.17 mL/d, *P *=* *0.095). The 2-year recurrence-free survival (RFS) was 0% for ALPPS and 35.7% for LAPS (*P *=* *0.009), whereas the 2-year overall survival for ALPPS and LAPS was 33.3% and 100.0% (*P *=* *0.052), respectively.

**Conclusions:**

LAPS tended to induce lower morbidity and FLR hypertrophy more slowly than ALPPS, with a comparable resection rate and better long-term RFS in HBV-related HCC patients.

## Introduction

Hepatectomy is a curative treatment for patients with hepatocellular carcinoma (HCC) [1]. For patients with low risk of microvascular invasion, liver resection can achieve surgical effects equivalent to liver transplantation [2]. However, insufficient future liver remnant (FLR) after surgery limits the possibility for patients to undergo hepatectomy, especially in patients with chronic liver diseases [3]. Patients with unresectable HCC with portal vein tumor thrombosis may be treated with chemoembolization but the median overall survival time is only 10.4 months [4]. Generally, an FLR/standard liver volume (SLV) ratio of > 30% after hepatectomy is considered to be safe in a normal liver. However, for a cirrhotic liver, an FLR/SLV ratio of >40% is needed [5]. He *et al*. [6] reported that the prevalence of liver cirrhosis in hepatitis B virus (HBV)-related HCC can escalate to an extent of ≤90%. Associating liver partition and portal vein ligation for staged hepatectomy (ALPPS) is an effective surgical method that can promote hypertrophy of the FLR, thereby increasing the resection rate of liver cancer [7].

ALPPS has a relatively high morbidity and mortality rate during the stage-1 operation [8]. Therefore, different modified ALPPS techniques have been proposed to improve the safety of the surgery. Conrad *et al.* [9] reported the use of laparoscopy for the stage-1 operation in 2012 and Machado *et al*. [10] reported the use of total laparoscopy for ALPPS surgery in the same year. The use of laparoscopic technology can significantly reduce abdominal adhesions and improve the safety of the stage-2 operation. To further reduce the trauma of the first operation, thermal ablation techniques were introduced into ALPPS surgery. Gall *et al*. [11] used radio-frequency-assisted liver partition with portal vein ligation for liver regeneration in five patients with colorectal liver metastasis and hepatectomy was successfully performed in all of these patients. Hong *et al*. [12] reported percutaneous microwave ablation liver partition and portal vein embolization for rapid liver regeneration in one patient with HCC who underwent right trisectionectomy on Day 14 after percutaneous microwave ablation liver partition and portal vein embolization. Gringeri *et al*. [13] proposed laparoscopic microwave ablation and portal vein ligation for staged hepatectomy (LAPS) in an HCC patient with alcoholic hepatopathy who underwent a stage-2 operation 10 days later. All of these modified ALPPS techniques can effectively promote liver hypertrophy with low morbidity and mortality rates. However, most of these studies were case reports, with the exception of the REBIRTH study, in which associating liver partition with portal vein ligation for staged hepatectomy assisted with radiofrequency was proven to be more effective than portal vein embolization (PVE) in increasing the FLR volume and surgical resection rate [14]. In addition, no research has compared the efficacy and oncological outcomes of modified ALPPS and classic ALPPS in the treatment of HBV-related HCC.

In this study, we report our experience with LAPS and compare its efficacy in promoting FLR regeneration, safety, and influence on long-term oncological outcome with ALPPS in patients with HBV-related HCC.

## Methods

### Patients

**Figure 1.**
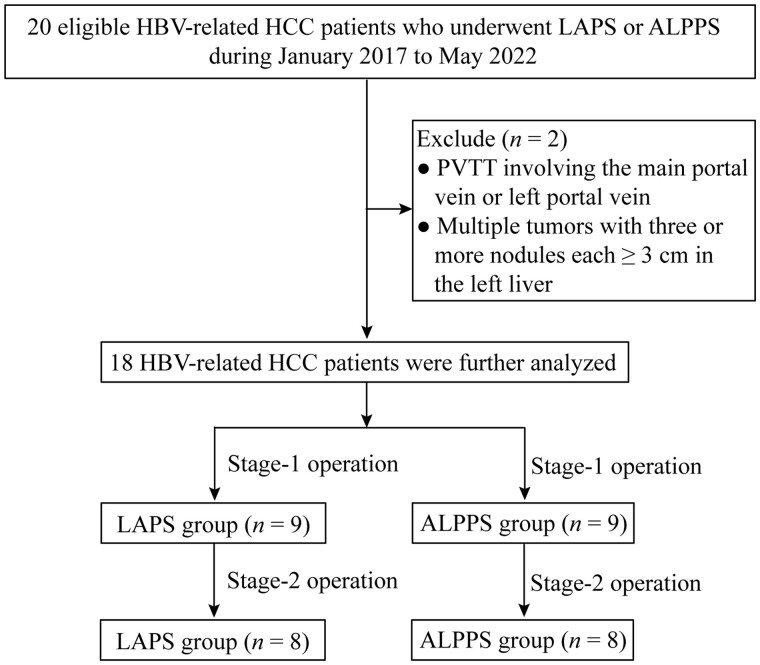
A flowchart demonstrating the study selection

Consecutive patients with HBV-related HCC who underwent LAPS or ALPPS in the First Affiliated Hospital, Sun Yat-sen University (Guangzhou, China) between January 2017 and May 2022 were retrospectively studied (Figure 1). All HBV-related HCC patients were diagnosed according to the guidelines of the European Association for the Study of the Liver (EASL) [15]. The research was approved by the hospital ethics committee and the protocol was in compliance with the Declaration of Helsinki.

The inclusion criteria for this study were as follows: (i) >18 years old; (ii) Child–Pugh class A disease; (iii) an FLR/SLV ratio of <30% in a normal liver and an FLR/SLV ratio of <40% in a cirrhotic liver; (iv) hepatitis B surface antigen positive; (v) primary or first recurrent HCC patients after radical resection or ablation; and (vi) no tumor lesions found on imaging 1 month after hepatectomy. The exclusion criteria were as follows: (i) Portal vein tumor thrombus involving the main portal vein or left portal vein; and (ii) multiple tumors with three or more nodules that were all ≥3 cm and in the left liver. All enrolled HCC patients with an FLR/SLV ratio of <30% in a normal liver or an FLR/SLV ratio of <40% in a cirrhotic liver could not tolerate extensive hepatectomy. In the early series, ALPPS was the preferred approach for our patients due to the learning curve. Since Gringeri *et al*. [13] reported LAPS, it has been the preferred treatment for our patients. The choice of treatment strategy was ultimately decided by our multidisciplinary team, including hepatobiliary surgeons, oncologists, radiologists, and anesthesiologists.

Before surgery, all patients provided written informed consent for their data to be used for research purposes. Clinical characteristics, including age, gender, preoperative liver function parameters, liver volume, tumor type, tumor number, tumor size, Barcelona Clinic Liver Cancer stage, and China liver cancer stage [16, 17], were included in the analysis. Post hepatectomy liver failure was defined according to the International Study Group of Liver Surgery classification [18]. The Clavien–Dindo criteria and the comprehensive complication index were used to grade post-operative complications [19, 20].

### Surgical procedures

#### LAPS

**Figure 2.**
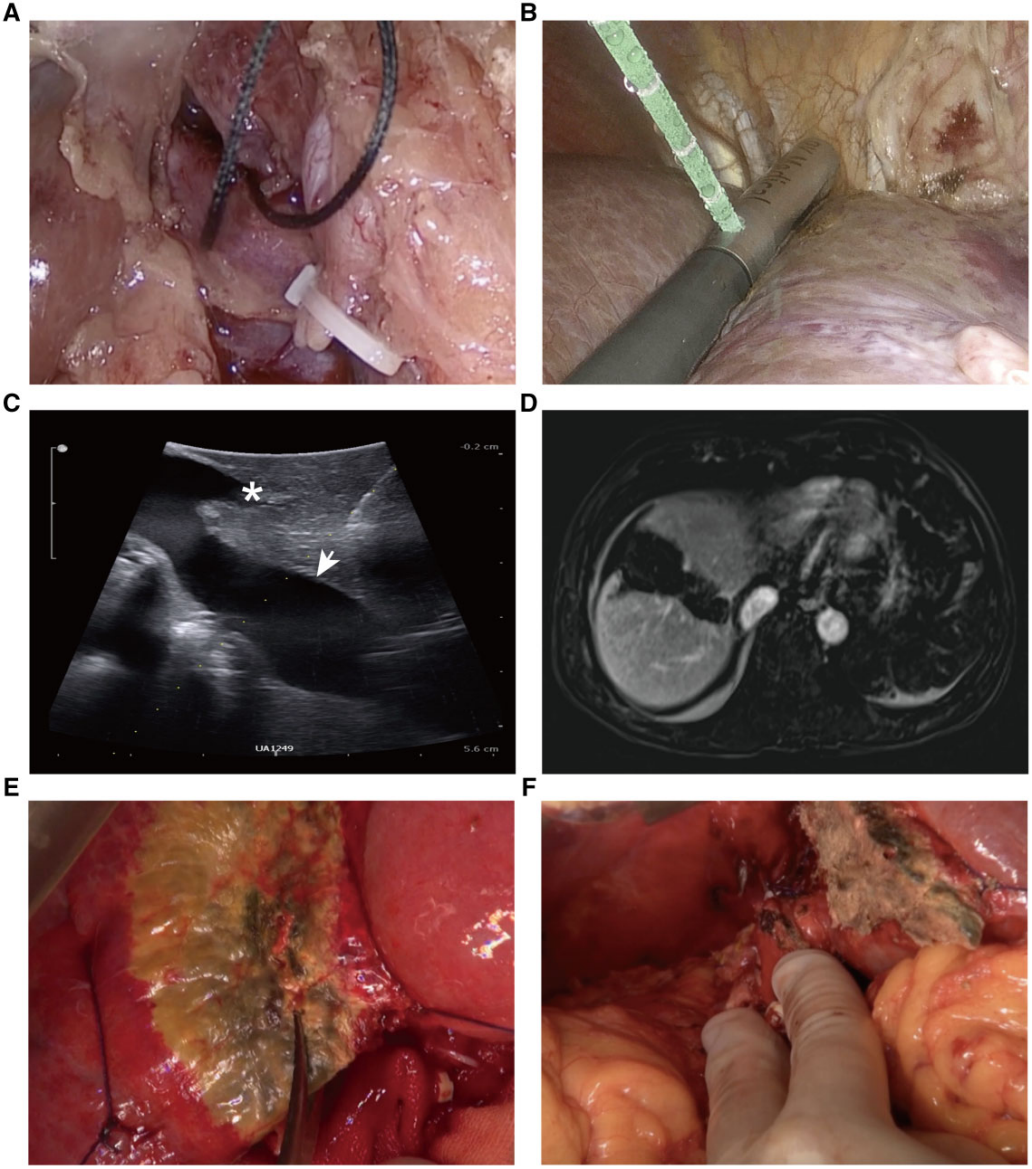
The procedure of laparoscopic microwave ablation and portal vein ligation for staged hepatectomy (LAPS). (A) Ligation of the right portal vein. (B) Microwave ablation guided by using ultrasound. (C) Microwave ablation zone (arrowhead: inferior vena cava; asterisk: middle hepatic vein). (D) Twelve days after stage-1 operation, MRI shows complete separation of the liver parenchyma. (E) Right hemi-hepatectomy was performed 15 days after the first operation. (F) Complete right hemihepatectomy.

The first LAPS was performed in April 2018. The patient was placed in the supine position and a 15-degree-feet-down position, and the surgeon stood on the right side of the patient. The camera was inserted through a 10-mm port below the umbilicus. Pneumoperitoneum was established with carbon dioxide at 12 mmHg. Two 12-mm ports were inserted 3 cm below the right midclavicular line and 5 cm below the xiphoid as the main working ports for performing laparoscopic ultrasound (LUS). Two 5-mm ports were inserted 3 cm below the right anterior axillary line and 3 cm below the left midclavicular line. After cholecystectomy, the right portal vein, located posterior to the right hepatic artery, was carefully separated from the common hepatic duct and ligated using 0 mersilk. The right hepatic artery was dissected and slung with 2-0 prolene sutures for future ligation in the stage-2 liver resection (Figure 2A). After dissecting the hepatic hilum, a four-way laparoscopic 8666-RF, rigid or flexible 4.3–10 MHz linear ultrasound transducer (BK Medical Holding Company, Inc., Herlev, Denmark) was used for scanning. The liver was scanned by using LUS, confirming that there was no tumor in the FLR, and anatomic techniques to trace the portal vein branches to the Couinaud segments. Three hepatic veins are shown from the confluence of the inferior vena cava. The transection plane was identified and marked by using an electrotome based on the relationship between the tumor and the middle hepatic vein. For a right hepatectomy or an extended right hemihepatectomy, the transection plane was on the right side of the middle hepatic vein and, for a right trisectionectomy, the plane was on the left side of the middle hepatic vein. Then, the microwave ablation antenna (Canyon Medical, Nanjing, China) was inserted into the parenchyma along the marked line from the liver capsule to 0.5 cm in front of the inferior vena cava, applying a 3-min ablation cycle (power output: 60 W) (Figure 2B). Parenchymal ablation commenced in a cranial-to-caudal fashion with a stitch distance of 2–3 cm (Figure 2C). No peritoneal drain or adhesion barrier films were placed. On the sixth to seventh day after the stage-1 operation, the volume of the FLR was estimated by using contrast-enhanced computed tomography (CT) or magnetic resonance imaging (MRI) (Figure 2D). For patients without fibrosis or cirrhosis, the FLR needed to be >30% of the SLV to undergo the stage-2 operation. For patients with fibrosis or cirrhosis, the FLR needed to be >40% of the SLV. If the FLR hypertrophy had not reached the anticipated size, CT/MRI was repeated every 7 days until 28 days after surgery.

In the stage-2 operation, a bilateral subcostal incision technique was selected. Intraoperative ultrasound was used to confirm that there was no tumor in the FLR and the liver parenchyma was transected along the ablation line using a harmonic scalpel (Figure 2E). The right hepatic artery, right hepatic bile duct, right portal vein, and right and middle hepatic veins were ligated and severed (Figure 2F). A drainage tube was placed under the right diaphragm.

#### ALPPS

In the stage-1 operation, bilateral subcostal incisions were made. The right branch of the portal vein was ligated using 0 mersilk and the right hepatic artery was encircled by using 2-0 prolene sutures. Then, parenchymal splitting was performed using a harmonic scalpel to the front of the inferior vena cava. In the stage-2 operation, the right hepatic artery, right hepatic bile duct, right portal vein, and right and middle hepatic veins were ligated and severed, and the right liver was completely removed. A drainage tube was placed under the right diaphragm.

### FLR assessment

The patient’s SLV was calculated according to the Urata formula based on the patient’s height and weight [21]. For the patients who underwent CT, the FLR was measured using Myrian software (Myrian XP Liver; Intrasense; France). For the patients who underwent MRI, the FLR was measured using the 3D volumetric post-processing workstation (VitreaCore version 6.5.5, Toshiba (Australia) Pty Ltd, North Ryde, Australia).

### Follow-up

The treatment response of patients was evaluated by using abdominal CT or MRI examination 1 month after the stage-2 operation. Thereafter, patients were followed up every 3 months for the first 2 years, every 6 months from 2 to 5 years, and annually after 5 years. The follow-up entailed contrast-enhanced CT of the upper abdomen, liver function test, and serum alpha fetoprotein detection. MRI of the abdomen was performed when necessary.

### Statistical analysis

Metric data are expressed as medians with ranges for normally or non-normally distributed data. The normally distributed data were compared by using Student’s *t*-test and the non-normally distributed data were compared by using the Wilcoxon rank-sum test. Categorical variables are described as rates and were compared by using the chi-square test or Fisher’s exact test. The Kaplan–Meier method with the log-rank test was used to compare overall survival (OS) and recurrence-free survival (RFS) between the two groups. All computations relied on standard software (SPSS Statistics v26.0; IBM, Chicago, IL). A two-sided *P *<* *0.05 was considered statistically significant.

## Results

### Patient characteristics

During the study period, 18 patients with HBV-related HCC underwent ALPPS (*n *=* *9) or LAPS (*n *=* *9) at the Centre of Hepato-Pancreato-Biliary Surgery of our hospital due to an inadequate FLR (Table 1). The median follow-up time of the 18 patients was 23.0 months (range, 4.0–67.0 months). Among 18 patients, 14 (77.8%) were male and 14 (77.8%) had primary HCC. Ten patients (55.6%, 10/18) had a single tumor. The median maximum tumor diameter was 82.0 mm (range, 26.0–127.0 mm). The liver function status of all patients was Child–Pugh class A. Based on the Barcelona Clinic Liver Cancer staging, the numbers of patients with stage A, B, and C diseases were nine (50.0%), four (22.2%), and five (27.8%), respectively. Based on the China liver cancer staging, the numbers of patients with stage Ib, IIa, IIb, and IIIa diseases were nine (50.0%), one (5.6%), four (22.2%), and four (22.2%), respectively. Of these 18 patients, 16 (88.9%) proceeded to curative hepatectomy (ALPPS, 88.9%, 8/9; LAPS, 88.9%, 8/9) (Table 2). Two of them did not undergo a second operation (ALPPS, 11.1%, 1/9; LAPS, 11.1%, 1/9) due to an insufficient increase in the FLR volume. In these cases, alternative treatments including transcatheter arterial chemoembolization were employed. There was no significant difference in the type of hepatectomy or the grade of liver fibrosis between the two groups (Table 1).

**Table 1.** Clinicopathological data of patients in the ALPPS and LAPS groups

Variable	Total (*n *=* *18)	ALPPS (*n *=* *9)	LAPS (*n *=* *9)	*P-*value
Median age (range), years	40.50 (24.0, 60.0)	38.00 (24.0, 55.0)	47.0 (28.0, 60.0)	0.251
Gender, *n* (%)				1.000
Male	14 (77.8)	7 (77.8)	7 (77.8)	
Female	4 (22.2)	2 (22.2)	2 (22.2)	
Median HBsAg level (IQR), IU/mL	563.5 (5.6, 2607.0)	250.0 (1.0, 1864.0)	582 (167.2, 4035.0)	0.449
HBV-DNA, *n* (%), IU/mL				1.000
≤100	10 (55.6)	5 (55.6)	5 (55.6)	
>100	8 (44.4)	4 (44.4)	4 (44.4)	
Tumor type, *n* (%)				1.000
Primary	14 (77.8)	7 (77.8)	7 (77.8)	
Recurrent	4 (22.2)	2 (22.2)	2 (22.2)	
Tumor number, *n* (%)				0.637
Single tumor	10 (55.6)	4 (44.4)	6 (66.7)	
Multiple	8 (44.4)	5 (55.6)	3 (33.3)	
Child–Pugh score 5, *n* (%)	18 (100.0)	9 (100.0)	9 (100.0)	NA
Portal hypertension, *n* (%)	3 (16.7)	2 (22.2)	1 (11.1)	1.000
Tumor size (IQR), mm	82.0 (26.0, 165.00)	82.0 (26.0, 165.00)	82.0 (29.0, 135.0)	0.468
AFP, *n* (%), μg/L				0.637
≤400	8 (44.4)	3 (33.3)	5 (55.6)	
>400	10 (55.6)	6 (66.7)	4 (44.4)	
BCLC staging, *n* (%)				0.689
A (early)	9 (50.0)	4 (44.4)	5 (55.6)	
B (intermediate)	4 (22.2)	3 (33.3)	1 (11.1)	
C (advanced)	5 (27.8)	2 (22.2)	3 (33.3)	
CNLC staging, *n* (%)				0.565
Ib	9 (50.0)	4 (44.4)	5 (55.6)	
IIa	1 (5.6)	1 (11.1)	0 (0.0)	
IIb	4 (22.2)	3 (33.3)	1 (11.1)	
IIIa	4 (22.2)	1 (11.1)	3 (33.3)	
SLV (Urata formula), median (range), mL	1,268.50 (1,035.00, 1348.00)	1,240.00 (1,043.00, 1346.00)	1,270.0 (1,035.0, 1348.0)	0.7775
Type of hepatectomy^a^				1.000
Right trisectionectomy, *n* (%)	5 (31.3)	3 (37.5)	2 (25.0)	
Right hemihepatectomy, *n* (%)	7 (43.8)	3 (37.7)	4 (50.0)	
Extended right hemihepatectomy, *n* (%)	4 (25.0)	2 (25.0)	2 (25.0)	
METAVIR grade of liver fibrosis, *n* (%)^a^				0.114
0 (no fibrosis/cirrhosis)	1 (16.3)	0 (0.0)	1 (12.5)	
1 (mild fibrosis)	5 (31.3)	1 (12.5)	4 (50.0)	
2 (moderate fibrosis)	5 (31.3)	4 (50.0)	1 (12.5)	
3 (severe fibrosis)	2 (12.5)	2 (25.0)	0 (0.0)	
4 (cirrhosis)	3 (18.8)	1 (12.5)	2 (25.0)	
Intrahepatic portoportal collaterals after stage-1 operation, *n* (%)	7 (38.9)	1 (11.1)	6 (66.7)	0.016

a One patient in the LAPS group and one patient in the ALPPS group did not complete the second stage and had no pathological diagnosis of cirrhosis. ALPPS, associating liver partition and portal vein ligation for staged hepatectomy; LAPS, laparoscopic microwave ablation and portal vein ligation for staged hepatectomy; BCLC, Barcelona Clinic Liver Cancer; CNLC, China liver cancer; IQR, inter quartile range. Data are expressed as median (range).

**Table 2.** Pre- and post-operative increases in FLR between ALPPS and LAPS

Variable	Total (*n *=* *18)	ALPPS (*n *=* *9)	LAPS (*n *=* *9)	*P*-value
Complete resection, *n* (%)	16 (88.9)	8 (88.9)	8 (88.9)	1.000
Interval between stage-1 and stage-2 operation, days^a^	14.0 (9.0, 28.0)	12.0 (9.0, 16.0)	15.0 (12.0, 28.0)	0.020
Before stage-1 operation				
FLR, mL	358.0 (247.0, 455.0)	350.0 (287.0, 412.0)	379.0 (247.0, 455.0)	0.707
FLR/SLV, %	29.0 (20.0, 36.0)	29.0 (22.0, 35.0)	30.0 (20.0, 36.0)	0.951
After stage-1 operation^a^				
FLR, mL	488.0 (321.0, 727.0)	493.0 (321.0, 685.0)	483.0 (383.0, 727.0)	0.853
FLR/SLV, %	42.0 (24.0, 55.0)	44.0 (24.0, 54.0)	38.0 (30.0, 55.0)	0.629
FLR increase, mL	154.0 (23.0, 287.0)	170.0 (23.0, 287.0)	136.0 (73.0, 272.0)	0.354
Increase rate of FLR, %	45.7 (19.3, 89.3)	58.1 (23.3, 84.7)	33.2 (19.3, 89.3)	0.296
Absolute KGR, mL/day	18.66 (2.30, 39.00)	24.29 (2.30, 39.00)	13.17 (6.13, 30.22)	0.095
Relative KGR, %/day	1.00 (0.0, 3.00)	2.00 (1.00, 3.00)	1.00 (1.00, 2.00)	0.132

a One patient in the LAPS group and one patient in the ALPPS group who did not complete the second stage were excluded. ALPPS, associating liver partition and portal vein ligation for staged hepatectomy; LAPS, laparoscopic microwave ablation and portal vein ligation for staged hepatectomy; FLR, future liver remnant; SLV, standard liver volume; KGR, kinetic growth rate. Data are expressed as median (range).

### Effect on volumetric changes in the liver

The data on preoperative baseline FLR volumes and post-operative FLR volume increase are listed in Table 2. In our study, the median time between stage-1 and stage-2 operations in the ALPPS group (12.0 days; range, 9.0–16.0 days) was shorter than that in the LAPS group (15.0 days; range, 12.0–28.0 days) (*P *=* *0.020). The speed of liver regeneration was expressed by the daily rate of hypertrophy. However, there was no difference in FLR volume increase (170.00 vs 136.00 mL, *P *=* *0.354) or absolute kinetic growth rate (24.29 vs 13.17 mL/day, *P *=* *0.095) between these two groups (**Table 2**).

### Perioperative safety

Compared with the ALPPS group, the LAPS group had less blood loss (50 vs 300 mL, *P *<* *0.001) during the stage-1 operation and a trend towards a lower overall comprehensive complication index (8.66 vs 35.87, *P *=* *0.054) (Table 3). There was no significant difference in red blood cell transfusion, overall post-operative complications (87.5% vs 62.5%), or overall liver failure (62.5% vs 75.0%) between the ALPPS and LAPS groups. The patients in the LAPS group had a significantly shorter median time from surgery to first passage of flatus (1.0 vs 3.0 days, *P *=* *0.005) and walking (2.0 vs 3.0 days, *P *=* *0.008) after the stage-1 operation than those in the ALPPS group. Additionally, the LAPS group also experienced a shorter duration for the first passage of flatus after the stage-2 operation (1.0 vs 3.0 days, *P *=* *0.015) than the ALPPS group did (Table 3).

**Table 3.** Operative data and post-operative complications of LAPS and ALPPS patients

Variable	Total (*n *=* *18)	ALPPS (*n *=* *9)	LAPS (*n *=* *9)	*P-*value
Stage-1 operation				
Operative time, min	280.0 (150.0, 635.0)	280.0 (150.0, 420.0)	280.0 (210.0, 635.0)	0.690
Blood loss, mL	150.0 (10.0, 1000.0)	300.0 (60.0, 1000.0)	50.0 (30.0, 100.0)	0.001
RBC transfusion, mL	0 (0, 600.0)	0 (0, 600.0)	0 (0.0, 0.0)	0.169
Post-operative complications, *n* (%)^a^				0.320
Grade I	8 (44.4)	4 (44.4)	4 (44.4)	
Grade II	4 (22.2)	3 (33.3)	1 (11.1)	
Grade IIIa	1 (5.6)	1 (11.1)	0 (0.0)	
Grade IIIb	0 (0.0)	0 (0.0)	0 (0.0)	
Grade IV	0 (0.0)	0 (0.0)	0 (0.0)	
Grade V	0 (0.0)	0 (0.0)	0 (0.0)	
Liver failure, *n* (%)^b^				0.311
Grade A	7 (38.9)	3 (33.3)	4 (44.4)	
Grade B	3 (16.7)	3 (33.3)	0 (0.0)	
Grade C	0 (0.0)	0 (0.0)	0 (0.0)	
CCI	8.66 (0, 44.70)	8.66 (0, 44.70)	8.66 (0, 20.90)	0.061
Time to first passage of flatus, days	2.0 (1.0, 4.0)	3.0 (2.0, 4.0)	1.0 (1.0, 3.0)	0.005
Time to walk, days	3.0 (1.0, 13.0)	3.0 (2.0, 13.0)	2.0 (1.0, 3.0)	0.008
Stage-2 operation^c^				
Operative time, min	215.0 (100.0, 757.0)	133.0 (100.0, 757.0)	225.0 (200.0, 690.0)	0.066
Blood loss, mL	500.0 (50.0, 1500.0)	300.0 (50.0, 900.0)	750.0 (200.0, 1500.0)	0.064
RBC transfusion, mL	0 (0, 900.0)	0 (0, 400.0)	0 (0, 900.0)	0.848
Post-operative complications, *n* (%)^a^				0.899
Grade I	4 (25.0)	2 (25.0)	2 (25.0)	
Grade II	3 (18.8)	2 (25.0)	1 (12.5)	
Grade IIIa	4 (25.0)	2 (25.0)	2 (25.0)	
Grade IIIb	0 (0.0)	0 (0.0)	0 (0.0)	
Grade IV	1 (6.3)	1 (12.5)	0 (0.0)	
Grade V	0 (0.0)	0 (0.0)	0 (0.0)	
Liver failure, *n* (%)^b^				1.000
Grade A	6 (37.5)	3 (37.5)	3 (37.5)	
Grade B	5 (31.3)	3 (37.5)	2 (25.0)	
Grade C	0 (0.0)	0 (0.0)	0 (0.0)	
CCI	14.79 (0, 46.20)	20.92 (0, 46.20)	4.33 (0, 33.50)	0.163
Time to first passage of flatus, days	2.0 (1.0, 5.0)	3.0 (2.0, 5.0)	1.0 (1.0, 4.0)	0.015
Time to walk, days	3.0 (2.0, 8.0)	4.0 (2.0, 8.0)	3.0 (2.0, 5.0)	0.205
Total operative time, min^c^	280.0 (150.0, 635.0)	270.0 (150.0, 360.0)	305 (240.0, 635.0)	0.268
Total blood loss, mL^c^	775.0 (300.0, 1900.0)	700.0 (300.0, 1900.0)	780.0 (300.0, 1700.0)	0.713
Total RBC transfusion, mL^c^	0 (0, 900.0)	150.0 (0, 400.0)	0 (0, 900.0)	0.432
Overall post-operative complications, *n* (%)				0.899
Grade I	4 (25.0)	2 (25.0)	2 (25.0)	
Grade II	3 (18.8)	2 (25.0)	1 (12.5)	
Grade IIIa	4 (25.0)	2 (25.0)	2 (25.0)	
Grade IIIb	0	0	0	
Grade IV	1 (6.3)	1 (12.5)	0	
Grade V	0	0	0	
Overall liver failure, *n* (%)				1.000
Grade A	7 (43.8)	3 (37.5)	4 (50.0)	
Grade B	4 (25.0)	2 (25.0)	2 (25.0)	
Grade C	0	0	0	
Overall CCI	25.24 (0, 65.62)	35.87 (8.66, 65.62)	8.66 (0, 47.10)	0.054
Hospital stays after stage-2 operation, days^c^	11.0 (9.50, 15.0)	12.5 (9.5, 17.0)	10.5 (7.0, 34.0)	0.527

ALPPS, associating liver partition and portal vein ligation for staged hepatectomy; LAPS, laparoscopic microwave ablation and portal vein ligation for staged hepatectomy; RBC, red blood cell; CCI, comprehensive complication index. The values of CCI are presented as median (range).

a Postoperative complication is graded according to the Clavien–Dindo classification of surgical complications.

b Liver failure is graded according to the International Study Group of Liver Surgery (ISGLS) classification.

c One patient in the LAPS group and one patient in the ALPPS group did not complete the second stage. Data are expressed as median (range).

### Oncological outcomes

**Figure 3.**
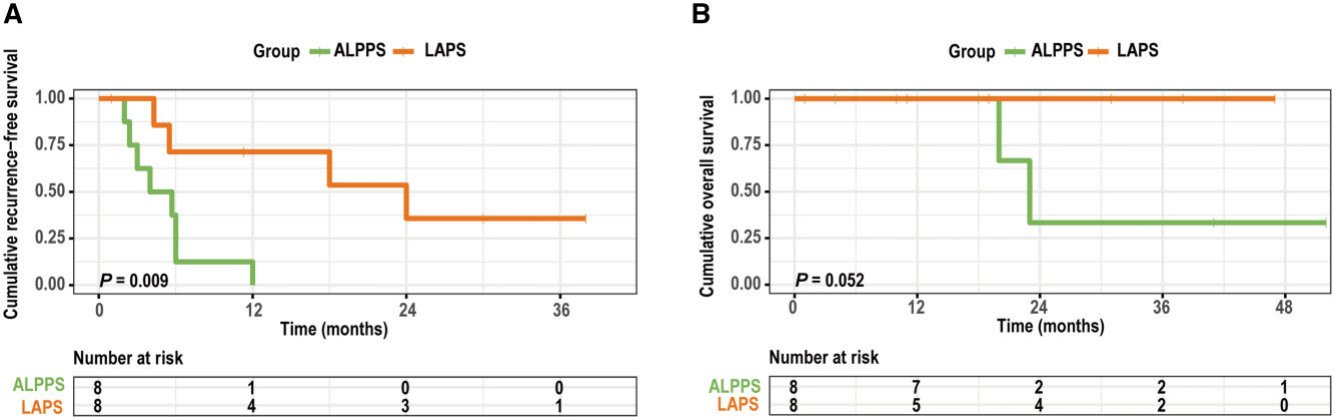
Survival outcome for LAPS and ALPPS. (A) Recurrence-free survival rate of patients after LAPS is slightly higher than that after ALPPS (*P *=* *0.009). (B) The overall survival of patients after LAPS is comparable to that of patients after ALPPS (*P *=* *0.052).

In the patients who completed the stage-2 operation, the median RFS for ALPPS and LAPS was 4.9 and 24.0 months, respectively. The 2-year RFS rates (Figure 3A) for ALPPS and LAPS were 0% and 35.7%, respectively (*P *=* *0.009). The median OS in the ALPPS group was 23.0 months, whereas the median OS in the LAPS group could not be calculated due to fewer deaths during the follow-up period. The 2-year OS rates (Figure 3B) for the ALPPS and LAPS groups were 33.3% and 100.0%, respectively (*P *=* *0.052).

## Discussion

Most reports on modified ALPPS are case reports and do not provide the long-term survival of patients. This study included a substantial number of patients with HBV-related HCC who underwent LAPS and compared these patients with those who underwent ALPPS from the same center. Our study showed that FLR hypertrophy and the resection rate were not different between the two groups. LAPS reduced the complications compared with ALPPS in the enrolled patients and the patients in the LAPS group recovered more quickly than those in the ALPPS group. In addition, LAPS was found to be superior to ALPPS with respect to RFS, but there was no difference in survival between the two groups.

PVE is the recommended treatment for the induction of FLR hyperplasia in many liver centers. However, liver regeneration takes a long time and 20%–30% of patients miss the opportunity for hepatectomy due to tumor progression [22–24]. ALPPS induces FLR hypertrophy in a short time and shows promising results in colorectal liver metastases. As reported, the FLR increase after the stage-1 operation reached 84% and the tumor resection rate was as high as 97% [25]. For patients with HBV-related HCC, concomitant liver fibrosis or cirrhosis might influence liver regeneration. Nevertheless, the FLR increase after the stage-1 operation still reached 56.8% and the completion rate of the stage-2 surgical resection reached 91% [26]. However, the high post-operative morbidity and mortality have limited the use of ALPPS. In recent years, different modified ALPPS methods have been proposed to improve safety, including minimally invasive methods in both stage-1 and stage-2 operations, partial splitting of the liver parenchyma, tourniquet partial ALPPS, and radiofrequency or microwave ablation-assisted ALPPS [13, 27–29].

Gringeri *et al*. [13] reported the successful treatment of a case of alcohol-related HCC with LAPS in 2015. In the same year, Cillo *et al*. [30] successfully applied LAPS to a patient with liver metastasis from colorectal cancer. In 2016, Chen *et al.* [31] applied microwave ablation to treat a patient with HBV-related HCC through laparotomy. In 2017, Wang *et al.* [29] applied radiofrequency ablation to separate the liver during the stage-1 operation and the resection rate of the stage-2 operation was 80%. However, the use of LAPS to induce FLR hypertrophy in HCC patients with chronic liver disease has not been reported. It was reported that the median interval from PVE to hepatectomy was 28 days (range, 21–45 days) [32], which was significantly longer than the 15 days required by LAPS in the present study. In our study, there was no difference in resection rates between the two groups, indicating that LAPS is a good alternative to ALPPS.

The incidence of major morbidity after ALPPS surgery was reported to be as high as 83% and the mortality within 90 days after surgery was as high as 48% [33]. Linecker *et al*. [34] reported that the occurrence of morbidity after the stage-1 operation was an independent risk factor that affected patient death within 90 days after surgery. However, when radiofrequency ablation was applied to separate the liver parenchyma during the stage-1 operation, there were no reports of bile leakage, bleeding, or abdominal infection after the operation [11]. All nine patients in our LAPS group had no severe morbidity or liver insufficiency. However, three patients in the ALPPS group had severe liver failure and one patient developed biliary leakage after the stage-1 operation and received percutaneous catheter drainage. Bleeding in the stage-1 operation of the LAPS group was significantly less frequent than that of the ALPPS group. There was no significant difference between the two groups in the incidence of morbidity and liver insufficiency after the stage-2 operation, whereas the overall comprehensive complication index tended to be lower in the LAPS group. LAPS may reduce post-operative morbidity.

**Figure 4.**
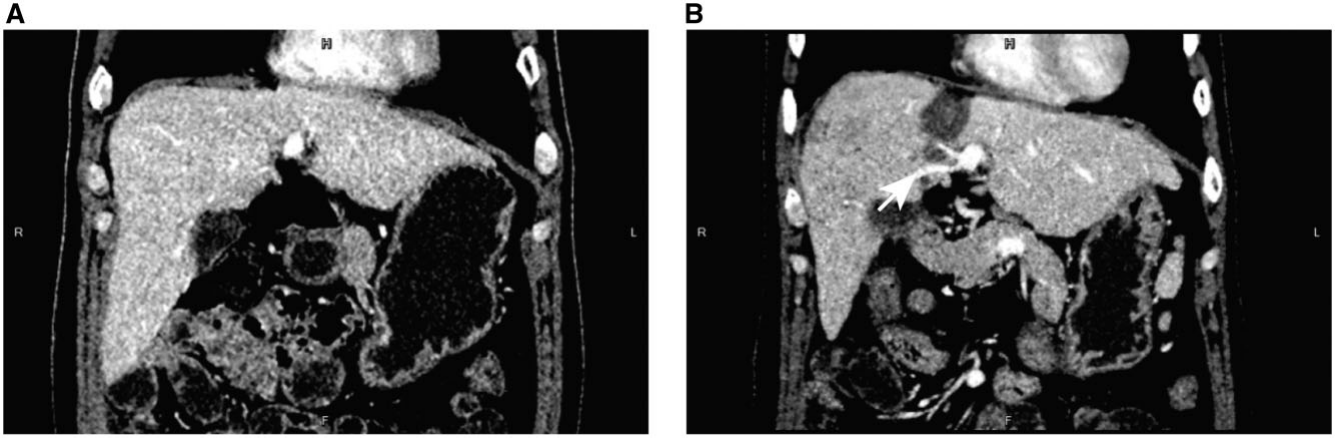
Contrast-enhanced computed tomography images show intrahepatic portoportal venous collateral vascular formation after liver partition and portal vein right branch ligation. (A) Before liver partition and portal vein right branch ligation. (B) Fourteen days after liver partition and portal vein right branch ligation (arrowhead: intrahepatic portoportal venous collateral vascular).

The severity of liver fibrosis and cirrhosis is an important factor associated with the FLR hypertrophy rate [26] and an imbalance in the severity of liver fibrosis and cirrhosis between the two groups may have led to differences in the rate of residual liver regeneration. In the study, the absolute kinetic growth rate of FLR in the ALPPS group was 24.29 mL/day, while it was 13.17 mL/day in the LAPS group, although there was no significant difference. In addition, we found that the rate of liver hypertrophy in the ALPPS group was faster than that in the LAPS group, even though the rate of severe fibrosis and cirrhosis was higher in the ALPPS group than in the LAPS group (37.5% vs 25.0%, *P *=* *0.114). The interval between the stage-1 and stage-2 operations was 15 days in the LAPS group, which was similar to the intervals reported by Gringeri *et al*. [13] and Cillo *et al*. [30] (10 and 15 days, respectively). The interval in the ALPPS group was 12.0 days, which was similar to what has been reported [26]. Changes in hemodynamics are an important factor affecting liver regeneration [35]. Studies have shown that intrahepatic portoportal collaterals are important factors affecting liver regeneration [36]. Patients with intrahepatic portoportal collaterals after the stage-1 operation took longer to reach a sufficient FLR volume for hepatectomy than patients without. In this study, the proportion of intrahepatic portoportal collaterals after the stage-1 operation in LAPS was significantly higher than that in ALPPS (66.7%, 6/9 vs 11.1%, 1/9, *P *=* *0.016). The interval between the two operations was 15.0 days for patients with intrahepatic portoportal collaterals and 12.0 days for patients without intrahepatic portoportal collaterals. This might be the reason for the slower hypertrophy of the FLR in the LAPS group (Figure 4).

Compared with percutaneous ablation, as reported by Hong *et al*. [12], laparoscopic ablation partition has several advantages. First, ablation can be performed directly along the intended resection line with accurate positioning. Second, the ablation range can be controlled to avoid excessive loss of normal liver tissue and reduce the risk of liver insufficiency after the stage-1 operation. Finally, the laparoscopic ablation needle is flexible, so even the liver tissue located below the diaphragm can be completely ablated, and thereby the completeness of the partition may be improved. In addition, ligation of the portal vein can be performed during LAPS. Although the same effect can be achieved by PVE in combination with percutaneous ablation, the cost-effectiveness should also be considered.

The study showed that LAPS may improve patient RFS compared with ALPPS. Liver partition by using ablation would better meet the ‘no-touch’ standard and avoid tumor dissemination caused by isolating and partitioning the liver. Thermal ablation can not only effectively kill tumor cells, but also release tumor antigens that can provoke an immune response, which might improve the prognosis for tumor patients [37]. We have previously tried to screen the immune-potentiating antigens and found that ficolin-3 was overexpressed in the serum of most HCC patients after radiofrequency ablation (RFA). Ficolin-3 might be a biomarker for RFA treatment efficacy and a potential target for HCC immunotherapy [38]. We will try to verify this in future studies that will include more LAPS cases. In addition, compared with the ALPPS group, patients in the LAPS group recovered faster after the stage-1 operation with a shorter time from surgery to first passage of flatus and walking, which was consistent with the enhanced recovery after surgery concept [39, 40]. If the procedure could be further improved to reduce adhesions after ablation and the stage-2 operation could also be performed laparoscopically, then the prognosis for patients in the LAPS group might be better, which needs to be verified in future clinical trials.

This study has several limitations. First, it was a retrospective study with a small sample size from a single center. Prospective randomized–controlled trials are still needed. Second, the rates of censored patients were high in both groups and were significantly higher in the ALPPS group than in the LAPS group. Third, it is possible that our results may not be applicable to patients with HCC in other countries because of different causes of liver disease. Additionally, the stage-1 operation of the LAPS group was performed laparoscopically; however, the same step of ALPPS in laparotomy is a limitation of this study. Laparoscopic ALPPS is the trend of the future, but it requires surgeons who are experienced with complex laparoscopy. Whether laparoscopic LAPS has fewer complications than laparoscopic ALPPS needs further study to confirm.

## Conclusions

LAPS tended to induce lower morbidity with a comparable resection rate and FLR hypertrophy and significantly prolonged RFS compared with ALPPS in HBV-related HCC patients. LAPS is a safe and effective treatment in HBV-related HCC patients with insufficient FLR.

## Authors’ Contributions

All authors contributed to the study conception and design. Data collection was performed by Z.B.C., W.X.X., and J.B.L. Analysis and interpretation were performed by Z.B.C., W.X.X., J.H.T., S.T.F., and S.Q.L. The draft of the manuscript was written by Z.B.C., W.X.X., and J.H.T. The manuscript was revised by M.K., J.H.T., and S.L.S. All authors read and approved the final manuscript.
